# Achilles Allograft Reconstruction for Ankle Multiligament Injuries in a Patient Presenting With Rotational Ankle Instability: A Surgical Technique

**DOI:** 10.1155/cris/9959031

**Published:** 2026-06-02

**Authors:** Morteza Gholipour, Amirhossein Salmannezhad, Fatemeh Abbasi

**Affiliations:** ^1^ Bone Joint and Related Tissues Research Center, Akhtar Orthopedic Hospital, Shahid Beheshti University of Medical Sciences, Tehran, Iran, sbmu.ac.ir; ^2^ School of Medicine, Shahid Beheshti University of Medical Sciences, Tehran, Iran, sbmu.ac.ir; ^3^ Department of Medicine, To.C., Islamic Azad University, Tonekabon, Iran, iauahvaz.ac.ir

## Abstract

The ankle comprises two main ligamentous complexes: the medial and lateral complex. In cases of chronic ankle instability following a sprain involving both complexes, rotational ankle instability can develop. We introduce a 25‐year‐old male with rotational ankle instability who reported having 20 episodes of “giving away” over the 6 months following an ankle sprain. Due to persistent instability, the patient was deemed a candidate for surgical reconstruction. An Achilles tendon allograft was utilized to reconstruct four ligaments—two from the lateral complex (the anterior talofibular ligament [ATFL] and calcaneofibular ligament [CFL]) and two from the medial complex (the tibiocalcaneal ligament and anterior tibiotalar ligament, components of the deltoid ligament). At the final follow‐up, the patient’s American Orthopedic Foot and Ankle Society (AOFAS) score was 89, the pain level was rated at 2 on the visual analog scale (VAS), and there was no significant restriction in the range of motion. While arthroscopic repair is the preferred option, an open surgical approach was chosen to adequately address all the ligamentous injuries, as the patient had two complete ruptures in addition to two partial tears.

## 1. Introduction

The ankle joint consists of three individual joints: talocalcaneal (subtalar), transverse tarsal (talocalcaneonavicular), and tibiotalar (talocrural) joint, which allow the ankle to move in all three planes (sagittal, transverse, and frontal) [1]. This complex joint is laterally stabilized by ligamentous support of the anterior talofibular ligament (ATFL), the posterior talofibular ligament (PTFL), and the calcaneofibular ligament (CFL). On the medial side, deltoid complex ligament, consisting of anterior tibiotalar ligament, tibiocalcaneal ligament, posterior tibiotalar ligament, and tibionavicular ligament provided the stability [2].

Ankle instability is a common consequence of ankle sprains. Lateral ankle sprains, which account for the majority of ankle sprain cases, are commonly led by a combination of plantar flexion and inversion of the ankle [3]. Injuries to the deltoid ligament, considered medial ankle sprain, can potentially coexist with chronic lateral ankle instability, reported as ranging between 40% and 72% [4, 5]. Studies demonstrated that 30%–70% of acute ankle sprains with inadequate management progress to chronic instability [6, 7]. In patients with chronic ankle instability, injury to the superior fascicle of the ATFL may lead to internal rotation of the talus, which can subsequently result in an overload injury to the deltoid ligament—a condition described as rotational ankle instability [8, 9]. Overall, rotational ankle instability is characterized by a concurrent injury involving the anterior fibers of the medial complex ligament and ATFL [8, 10].

While combined arthroscopic repair of lateral and medial ligaments for rotational ankle instability has been described, these techniques primarily utilize suture anchor‐based repair of native tissue [11–13]. Allograft reconstruction for chronic ankle instability has been reported for isolated lateral ligament injuries, but its application to simultaneous medial and lateral multiligament reconstruction remains underreported [14–16]. Furthermore, existing combined reconstruction techniques typically employ separate grafts or multiple anchor points for each ligament [12, 13]. To our knowledge, this is the first detailed technical description of using a single, continuous Achilles tendon allograft to reconstruct four ligaments—two from the lateral complex (ATFL and CFL) and two from the medial complex (tibiocalcaneal and anterior tibiotalar ligaments)—in a patient with rotational ankle instability.

## 2. Case Report

**Figure 1:**
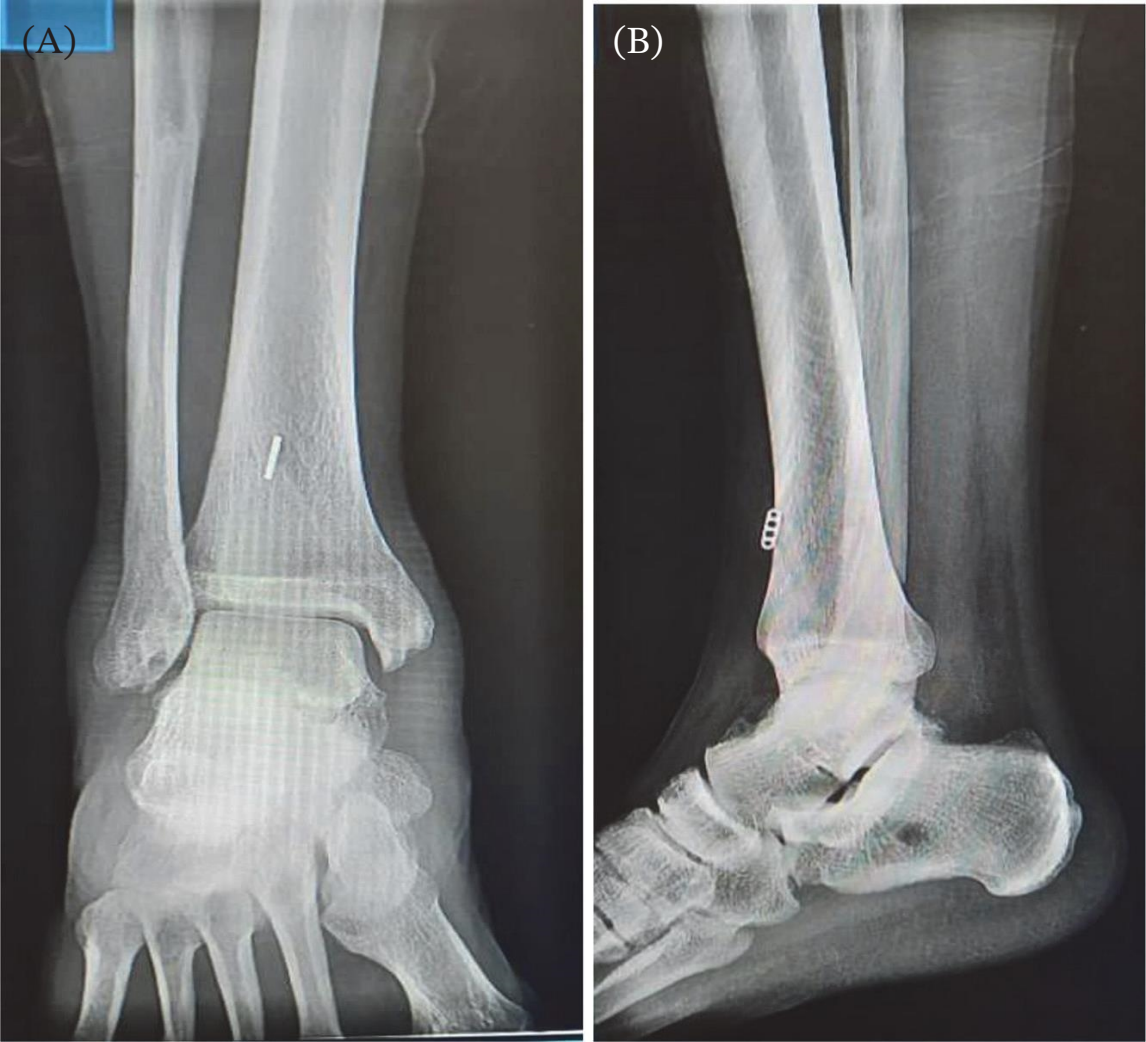
Standard X‐rays. Anteroposterior view (A) and lateral view (B) show no evidence of fracture.

A 25‐year‐old male presented to the hospital with complaints of frequent lateral ankle giving away. He claimed a history of falling from a height of ~3 m 6 months prior, resulting in a pure ankle dislocation. At that time, a closed reduction was performed, followed by 1 month of immobilization in a short leg cast and 6 months of physiotherapy focusing on strengthening the peroneal, tibialis posterior, and tibialis anterior muscles. According to the patient, he experienced nearly 20 episodes of ankle torsion during the subsequent months. On physical examination, the anterior drawer test was positive accompanying pain in the medial gutter of the ankle. Furthermore, valgus and pronation stress tests elicited pain. Reviewing the X‐ray on anteroposterior and lateral views, no evidence of fracture was seen (Figure 1). The MRI revealed partial tears in the syndesmosis ligament (anteroinferior tibiofibular ligament [AITFL]), CFL, and tibiocalcaneal ligament, as well as complete rupture of ATFL and anterior tibiotalar ligament.

## 3. Surgical Technique

**Figure 2:**
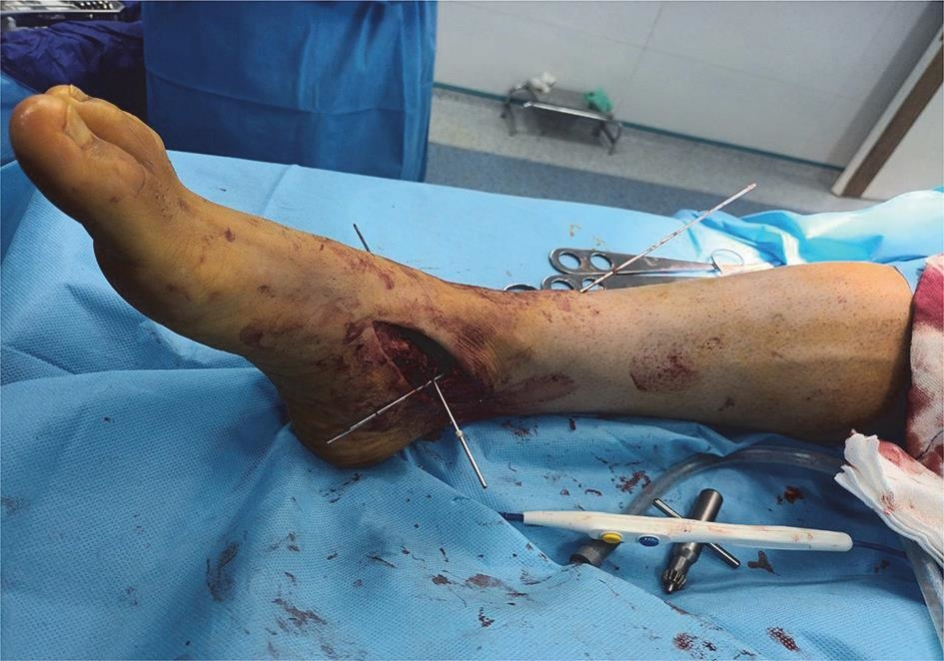
Two K‐wires were inserted: one from the tip of the medial malleolus to the anteromedial tibia; one from the deltoid ligament insertion site to the insertion site of the ATFL. A third K‐wire was also inserted from tibiocalcaneal ligaments to CFL, which has not been shown in this figure.

**Figure 3:**
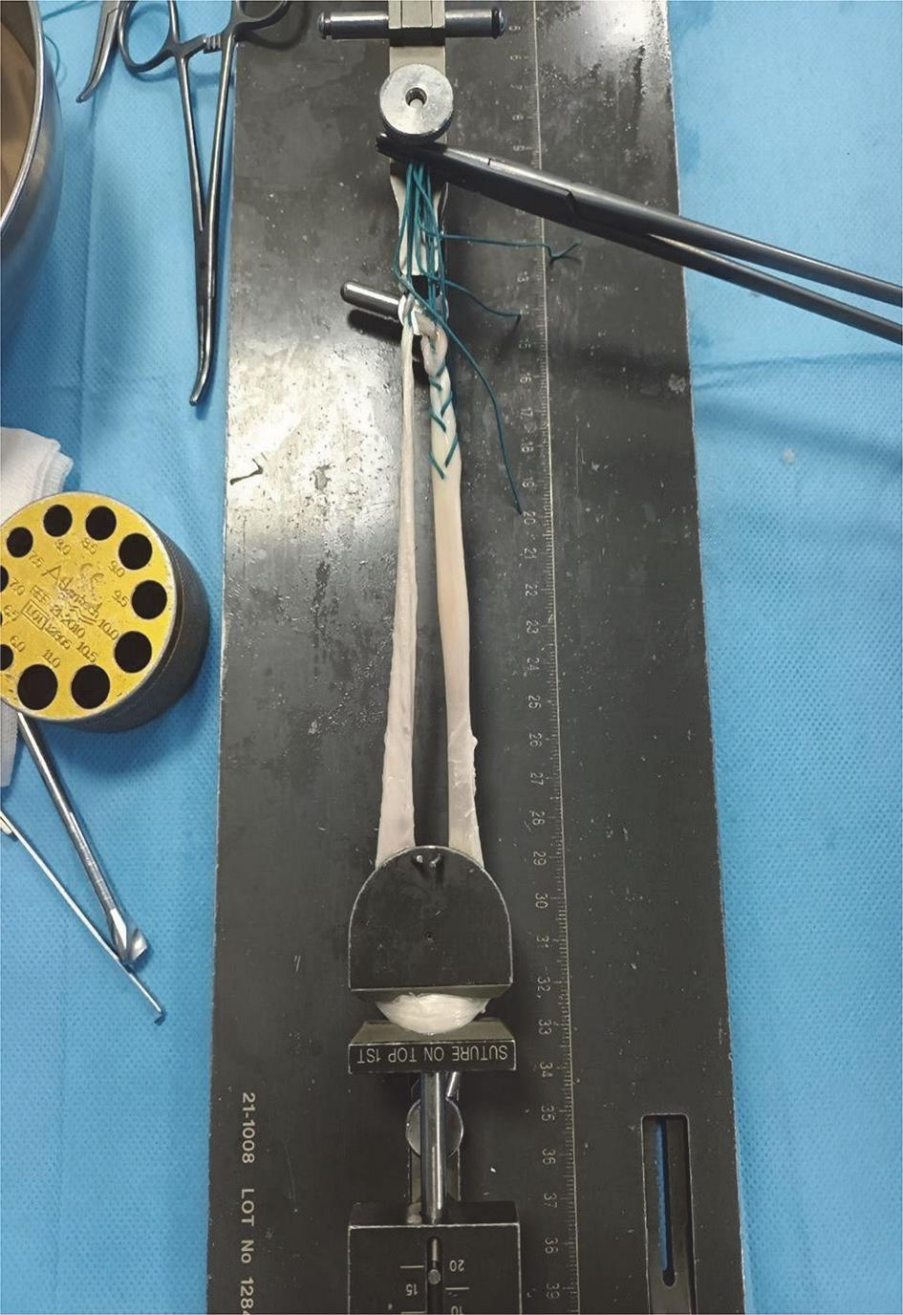
Tensile strength testing after preparing the Achilles tendon allograft.

**Figure 4:**
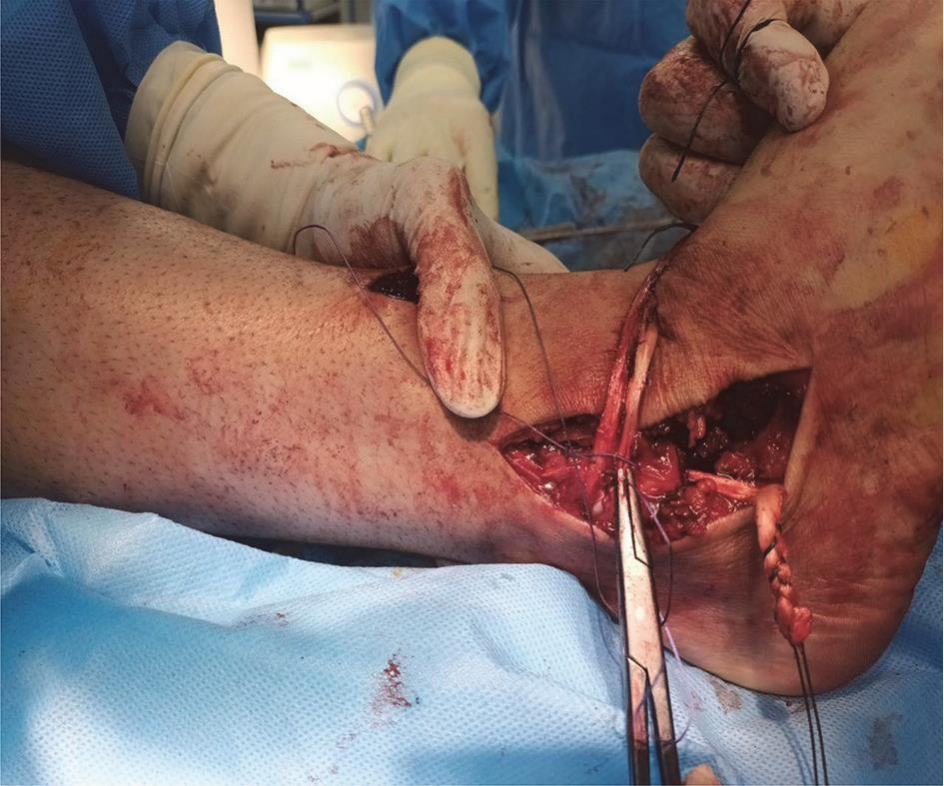
Two heads of allograft passed through the two tunnels created in fibula, then were sutured under modified tension.

Prior to surgery, ligaments and capsules were explored through arthroscopy. The patient was positioned supine with a high‐thigh tourniquet applied and underwent spinal anesthesia. Preoperative prepping and draping were performed in standard sterile fashion. A medial incision was made, extending from the tip of the medial malleolus to the talonavicular joint (~8 cm), to expose the anatomical insertions of the deltoid and posterior talotibial ligaments. Given that reconstruction of the medial ligaments (talar and calcaneal components) was planned, this incision was designed to reduce soft tissue tension and minimize the risk of tissue necrosis and subsequent wound complications. Through an anterolateral approach, a second incision was made from the anterior fibular region to the base of the 4th metatarsal, providing access to the anterior talofibular and CFLs. Afterward, three guide K‐wires were inserted: one through the tip of medial malleolus to the anteromedial tibia; one from deltoid ligament’ insertion site to that of the ATFL; and one from tibiocalcaneal ligaments to CFL (Figure 2). We utilized a single Achilles tendon allograft measuring ~20 cm in length and 3 cm in width. After preparation, the graft was configured into a construct ~50 cm in length and 6 mm in diameter. (Figure 3). The folded end of the graft was secured and fixed using an Endobutton. The graft was then passed in a spiral configuration through anatomically created bone tunnels corresponding to the native ligament structures of the ankle. On the medial side, the graft was passed through a canal created across the medial malleolus, with one limb directed to the talus and the other to the calcaneus. On the lateral side, two canals were created in fibula utilizing a 6 mm reamer: one from tip of the fibula to the posterior aspect; the other from posterosuperior to anteroinferior aspect of the fibula (Figure 4). The two free limbs of the allograft were then tunneled through these canals and stabilized under appropriate tension by sutures. At each intersection point of the graft, fixation was reinforced using No. 2 FiberWire sutures (Stryker), secured with six knots. After achieving appropriate ligamentous tension, the lateral end of the graft was secured using a Krackow suture technique and sutured to the remaining tendon. Additionally, a 6 mm bioabsorbable screw (Stryker) was used for fixation at the talar level. To further enhance construct stability, the lateral retinaculum was repaired. The graft diameter matched the tunnel diameter (6 mm), ensuring secure fixation without risk of loosening. Postoperatively, a short leg plaster was applied, and the patient was instructed to offload the affected limb for 6 weeks. Despite the partial tear of the AITFL, the syndesmotic joint was assessed to be stable and intact, which led the surgical team not to surgically address the injury during the initial procedure. During postoperative recovery, the joint remained stable with no clinical or radiographic signs of instability (medial clear space < 4 mm, normal talocrural angle, and no talar tilt); therefore, a nonsurgical approach was continued.

## 4. Follow‐Up

The patient was followed up at 6 weeks, 3 months, and 1 year postoperatively. At the final follow‐up, the patient had 10° of dorsiflexion, 20° of plantarflexion, and full range of motion in both inversion and eversion. The American Orthopedic Foot and Ankle Society (AOFAS) score was 89. Pain was assessed using the visual analog scale (VAS), which indicated a pain level of 2 out of 10.

## 5. Discussion

Diagnosis of rotational ankle instability, which affects both the lateral and medial complexes, can be challenging during physical examination, as the patients often exhibit various symptoms [8]. In this regard, arthroscopic interventions are highly useful to identify and treat the damaged ligaments [8, 11]. In this case, based on the typical clinical presentation, the patient’s history of previous injury, MRI findings, and arthroscopic evaluation, the diagnosis of rotational ankle instability was established.

Ankle sprains, both acute and chronic, are initially managed by nonsurgical interventions, such as physiotherapy, applying splints, and sport‐specific activities [17–19]. However, in cases suffering from chronic instability after sprain, surgical management is crucial to prevent intra‐articular complications such as posttraumatic osteoarthritis (PTOA) and degenerative arthritis [20, 21]. The decision to proceed with open surgery in this case was based on intraoperative findings during diagnostic arthroscopy. Complete ligamentous retraction, poor tissue quality, and the chronic nature of the injury precluded primary arthroscopic repair [22–24]. While arthroscopic repair has demonstrated comparable or superior outcomes to open repair for chronic ankle instability when adequate tissue quality exists, the literature supports conversion to open surgery when native ligament tissue is insufficient for repair [22, 24–26]. Arthroscopic techniques are most successful when ligament stumps can be identified and reattached to their anatomic footprints using suture anchors [13]. In this case, the extensive retraction and poor tissue quality made anatomic reattachment impossible arthroscopically, necessitating open reconstruction.

The modified Broström procedure, considered the gold standard for chronic lateral ankle instability, requires adequate native ligament tissue for imbrication and repair [26, 27]. This technique is contraindicated when ligament tissue quality is poor, when there is extensive medial and lateral ligamentous rupture, or in revision settings [25, 27, 28]. In this case, the presence of severe ligament insufficiency affecting both the lateral complex (ATFL and CFL) and medial complex (tibiocalcaneal and anterior tibiotalar ligaments) with poor tissue quality made primary repair unfeasible. The literature identifies several specific contraindications to the modified Broström procedure: generalized ligamentous laxity (Beighton score ≥4–5), high BMI, long‐standing ligamentous insufficiency, and inadequate ligament substance [27, 29, 30]. When these conditions exist, anatomic reconstruction with tendon graft is recommended to maintain physiological joint kinematics while providing adequate stability. Table 1 summarizes available surgical options for combined medial–lateral ankle ligament reconstruction.

**Table 1:** A summary of the surgical options for combined medial–lateral ankle ligament reconstruction.

Technique	Graft type	Advantages	Disadvantages	Indications	References
Modified Broström (open/arthroscopic)	None (primary repair)	Preserves native tissue; no donor site morbidity; maintains ROM; faster recovery; 85%–95% success rate	Requires adequate tissue quality; contraindicated in GJH, high BMI, poor tissue quality	First‐line for adequate ligament tissue; isolated lateral instability	[22–24]
Anatomic reconstruction—autograft	Gracilis, semitendinosus, peroneus brevis	Superior tissue integration; faster healing (11.2 months); better revascularization; no disease transmission risk	Donor site morbidity; longer operative time (85.5 min); limited graft length for multiligament reconstruction	Poor tissue quality; revision surgery; multiligament injuries with a single graft source	[13, 25–28]
Anatomic reconstruction—allograft	Achilles, semitendinosus, gracilis	No donor site morbidity; adequate length for multiligament reconstruction; shorter operative time (58.1 min); comparable outcomes to autograft	Longer healing time (13.5 months); theoretical disease transmission risk; higher cost; slower revascularization	Multiligament reconstruction requiring a long graft; avoidance of donor site morbidity; combined medial–lateral reconstruction	[13, 26, 28, 30]
Combined arthroscopic repair (medial + lateral)	None (suture anchors)	Minimal soft tissue dissection; addresses rotational instability; comparable outcomes to isolated lateral repair; lower wound complications	Requires identifiable ligament tissue; technically demanding; limited long‐term data	Rotational ankle instability with adequate tissue for anchor fixation	[29, 31]
Open combined reconstruction (medial + lateral)	Autograft (separate grafts)	Addresses all components of instability; proven outcomes; allows direct visualization	Multiple incisions; longer recovery; requires multiple graft sources or harvest sites	Chronic combined MCL–LCL injuries; rotational instability with poor tissue quality	[15, 32]
Nonanatomic tenodesis	Peroneus brevis (Evans, Chrisman‐Snook)	Strong construct; historical use	Restricts ROM; alters biomechanics; higher nerve injury risk; inferior long‐term outcomes	Rarely indicated; salvage procedures only	[24, 33]

In this case, an Achilles tendon allograft was selected due to insufficient native tissue length for reconstruction of all four ligaments and the need to avoid donor site morbidity. The Achilles tendon provides adequate length (typically 15–20 cm) and width to restore anatomical structures of both the lateral complex (ATFL and CFL) and medial complex (tibiocalcaneal and anterior tibiotalar ligaments) using a single, continuous graft [15, 16]. While autograft reconstruction demonstrates superior tissue integration and faster healing times (11.2 vs. 13.5 months), the clinical outcomes between autograft and allograft are comparable, with both achieving Karlsson‐Peterson scores >80 points and patient satisfaction rates >92% [15, 34, 35]. The systematic review by Spennacchio et al. [15] found no significant difference in validated outcome measures between autograft and allograft for lateral ligament reconstruction.

The key advantage of allograft in this multiligament scenario is the availability of sufficient graft length without requiring multiple autograft harvest sites, which would increase operative time and morbidity [34]. Biomechanical studies confirm that Achilles tendon grafts possess load‐to‐failure values (>400 N) that exceed those of native ATFL (231 ± 129 N) and CFL (307 ± 142 N), providing adequate strength for reconstruction [36]. While allograft demonstrates slower revascularization and higher T2 values on MRI compared to autograft, these differences do not translate to clinically significant outcome disparities at midterm follow‐up.

This study is limited by a relatively short follow‐up period, as a minimum follow‐up of 2 years is often considered necessary to assess late failure, recurrent instability, or the development of early PTOA.

## 6. Conclusion

Herein, we presented a case experiencing persistent instability with multiligament injuries, which was considered an ideal candidate for surgical treatment. MRI findings revealed tears in four ligaments (two complete and two partial), presenting challenges for arthroscopic repair. Although arthroscopic repair is the optimal option, an open surgical approach was chosen to adequately reconstruct all the ligamentous injuries.

## Funding

The authors have nothing to report.

## Ethics Statement

According to the institutional protocol of Shahid Beheshti University of Medical Sciences and Akhtar Orthopedic Hospital, Institutional Review Board (IRB) approval is not required for single‐patient case reports or technical notes. The described surgical procedure was performed as part of routine clinical management. Written informed consent was obtained from the patient for both the surgical treatment and the publication of this case report and accompanying images.

## Consent

The patient provided written informed consent for the publication of this case report, including all associated images and any relevant clinical details, ensuring a full understanding of the implications of sharing their medical information.

## Conflicts of Interest

The authors declare no conflicts of interest.
